# Brain Responses to Noxious Stimuli in Patients With Chronic Pain: A Systematic Review and Meta-analysis

**DOI:** 10.1001/jamanetworkopen.2020.32236

**Published:** 2021-01-05

**Authors:** Anna Xu, Bart Larsen, Alina Henn, Erica B. Baller, J. Cobb Scott, Vaishnavi Sharma, Azeez Adebimpe, Allan I. Basbaum, Gregory Corder, Robert H. Dworkin, Robert R. Edwards, Clifford J. Woolf, Simon B. Eickhoff, Claudia R. Eickhoff, Theodore D. Satterthwaite

**Affiliations:** 1 Department of Psychiatry, University of Pennsylvania, Philadelphia; 2 Department of Psychiatry, Psychotherapy and Psychosomatics, Medical Faculty, RWTH (Rheinisch-Westfälische Technische Hochschule) Aachen University, Aachen, Germany; 3 Department of Psychiatry, Massachusetts General Hospital, Boston; 4 Department of Psychiatry, Harvard University, Boston, Massachusetts; 5 VISN4 Mental Illness Research, Education, and Clinical Center at the Corporal Michael J. Crescenz VA (Veterans Affairs) Medical Center, Philadelphia, Pennsylvania; 6 Department of Anatomy, University of California, San Francisco; 7 Department of Anesthesiology and Perioperative Medicine, University of Rochester School of Medicine and Dentistry, Rochester, New York; 8 Department of Anesthesiology, Perioperative and Pain Medicine, Brigham and Women’s Hospital, Harvard Medical School, Boston, Massachusetts; 9 FM Kirby Neurobiology Center, Boston Children’s Hospital, Boston, Massachusetts; 10 Department of Neurobiology, Harvard Medical School, Boston, Massachusetts; 11 Institute of Systems Neuroscience, Medical Faculty, Heinrich-Heine University, Düsseldorf, Germany; 12 Institute of Neuroscience and Medicine, Brain and Behaviour Sections, Research Centre Jülich, Jülich, Germany; 13 Institute of Clinical Neuroscience and Medical Psychology, Medical Faculty, Heinrich-Heine-University, Düsseldorf, Germany

## Abstract

**Importance:**

Functional neuroimaging is a valuable tool for understanding how patients with chronic pain respond to painful stimuli. However, past studies have reported heterogenous results, highlighting opportunities for a quantitative meta-analysis to integrate existing data and delineate consistent associations across studies.

**Objective:**

To identify differential brain responses to noxious stimuli in patients with chronic pain using functional magnetic resonance imaging (fMRI) while adhering to current best practices for neuroimaging meta-analyses.

**Data Sources:**

All fMRI experiments published from January 1, 1990, to May 28, 2019, were identified in a literature search of PubMed/MEDLINE, EMBASE, Web of Science, Cochrane Library, PsycINFO, and SCOPUS.

**Study Selection:**

Experiments comparing brain responses to noxious stimuli in fMRI between patients and controls were selected if they reported whole-brain results, included at least 10 patients and 10 healthy control participants, and used adequate statistical thresholding (voxel-height *P* < .001 or cluster-corrected *P* < .05). Two independent reviewers evaluated titles and abstracts returned by the search. In total, 3682 abstracts were screened, and 1129 full-text articles were evaluated.

**Data Extraction and Synthesis:**

Thirty-seven experiments from 29 articles met inclusion criteria for meta-analysis. Coordinates reporting significant activation differences between patients with chronic pain and healthy controls were extracted. These data were meta-analyzed using activation likelihood estimation. Data were analyzed from December 2019 to February 2020.

**Main Outcomes and Measures:**

A whole-brain meta-analysis evaluated whether reported differences in brain activation in response to noxious stimuli between patients and healthy controls were spatially convergent. Follow-up analyses examined the directionality of any differences. Finally, an exploratory (nonpreregistered) region-of-interest analysis examined differences within the pain network.

**Results:**

The 37 experiments from 29 unique articles included a total of 511 patients and 433 controls (944 participants). Whole-brain meta-analyses did not reveal significant differences between patients and controls in brain responses to noxious stimuli at the preregistered statistical threshold. However, exploratory analyses restricted to the pain network revealed aberrant activity in patients.

**Conclusions and Relevance:**

In this systematic review and meta-analysis, preregistered, whole-brain analyses did not reveal aberrant fMRI activity in patients with chronic pain. Exploratory analyses suggested that subtle, spatially diffuse differences may exist within the pain network. Future work on chronic pain biomarkers may benefit from focus on this core set of pain-responsive areas.

## Key Points

**Question:**

Do the brains of patients with chronic pain respond differently to noxious stimuli?

**Findings:**

This systematic review and meta-analysis of 37 experiments from 29 unique articles including 944 participants found that patients with chronic pain were not associated with significant differential responses to noxious stimuli that induce pain compared with healthy controls.

**Meaning:**

Chronic pain does not appear to be associated with consistent marked alterations in the brain’s response to noxious stimuli.

## Teaser

This meta-analysis assesses differential brain responses to noxious stimuli in patients with chronic pain using functional magnetic resonance imaging while adhering to current best practices for neuroimaging meta-analyses.

## Introduction

Chronic pain is one of the most common and debilitating medical conditions worldwide,^1,2^ but existing treatments have modest efficacy, limited tolerability, and important safety risks.^3^ Neuroimaging has been increasingly used to investigate brain responses to noxious stimuli in patients with chronic pain, in the hope of finding an imaging signature that can accelerate the development of novel therapeutics.^3,4,5,6,7^ However, individual experiments are frequently underpowered, prone to false-positive findings, and diverse in the analytical pipelines and experimental designs used. These factors produce considerable heterogeneity in reported results^8,9,10,11,12,13,14,15^ and necessitate synthesizing results across experiments to identify consistent, systematic alterations of brain responses in chronic pain.^8,14,16,17,18,19^

Neuroimaging meta-analyses provide a powerful strategy to identify convergent brain regions altered in pain processing in chronic pain.^8,13,14^ However, existing meta-analyses examining differential brain responses to patients with chronic pain have yielded inconsistent findings, likely owing to differences in meta-analytic approaches used.^20,21,22,23^ For example, several meta-analyses have reported aberrant activity in the cingulate cortex,^20,21,22^ insula,^20,21,22^ and secondary somatosensory cortex,^20,21,22^ but others report aberrant activity in additional areas such as the cerebellum,^21,22^ primary somatosensory cortex,^22^ prefrontal cortex,^22^ intraparietal lobule,^22^ thalamus,^22^ and supplementary motor area.^22^ In addition, 1 meta-analysis^23^ found no differences in patients with chronic pain compared with healthy control participants. By adhering to new recommendations for best practices in neuroimaging meta-analyses,^13,14^ the present study sought to address 3 key limitations of prior work that may have contributed to such inconsistent findings.

First, most prior meta-analyses have compared patient and control participant responses to noxious stimuli across experiments^20,21,22^ by contrasting meta-analytic maps that are calculated separately for experiments with only patients and those with only healthy controls. However, this between-experiment contrast confounds differences in experimental design with patient group because experiments studying patients often use different functional magnetic resonance imaging (fMRI) tasks and pain stimulation procedures than studies of controls.^23^ Focus on within-experiment comparisons between patients and controls, where the noxious stimuli and imaging procedures used in both groups are identical, is a clear alternative that controls for variance in experimental procedures that would otherwise be present in between-experiment analyses. To our knowledge, no comprehensive meta-analysis of chronic pain has taken this approach; prior studies using this approach have only focused on specific conditions.^24,25,26^

Second, meta-analyses may introduce a source of bias by including experiments focused on specific regions of interest (ROI).^20^ Including such experiments that restrict analyses to specific brain areas may introduce substantial bias toward finding results in those regions.^13,14^ Current guidelines emphasize the importance of only including experiments reporting whole-brain results.^13,14^ This is particularly relevant for studies of chronic pain, in which ROI-based approaches have been commonly used.

Last, differences in statistical significance thresholds used to account for multiple comparisons introduce another critical source of variability. Within the past 10 years, high-profile reports^9,11,12^ have documented the failures of many widely used methods for controlling type I error in fMRI, leading to a large number of false-positive findings in the existing literature. In neuroimaging meta-analyses, this problem can be present at 2 levels. First, including individual experiments that do not adequately control for type I error will inflate apparent differences between patients and controls in the meta-analytic results. Second, because imaging meta-analyses compare hundreds of thousands of locations across the brain, meta-analyses themselves also need to rigorously control for multiple comparisons.^13,14,27^ However, existing meta-analyses have not consistently applied contemporary recommendations for type I error control at both levels.

We address these limitations in a preregistered meta-analysis focused on within-experiment comparisons, ensuring that differences due to patient group were not confounded with differences due to experimental design. Next, we reduced the potential for regional bias by requiring experiments to report whole-brain results. Finally, we only included experiments that applied some type I error control and then rigorously corrected for multiple comparisons in the meta-analysis itself. By addressing these limitations, we evaluated whether robust differences in brain responses to noxious stimuli between patients with chronic pain and healthy controls existed and were replicable across experiments.

## Methods

We conducted a systematic review and meta-analysis that followed the Preferred Reporting Items for Systematic Reviews and Meta-analyses (PRISMA) reporting guideline^28^ and field-standard guidelines for meta-analyses.^13,14^ The following procedures and analyses conducted in this meta-analysis were preregistered on PROSPERO. This study was deemed exempt from ethics approval by the University of Pennsylvania because only nonidentifiable summary statistics from published data were used.

Briefly, we searched for experiments published from January 1, 1990, to May 28, 2019, using PubMed/MEDLINE, EMBASE, Web of Science, Cochrane Library, PsycINFO, and SCOPUS. We used the following search terms: (*fMRI* or *functional magnetic resonance imaging* or *BOLD* or *brain mapping*) AND (*pain* or *noxious* or *nociception*) AND (*patients* or *neuropathic* or *chronic pain* or *hyperalgesia* or *allodynia*) OR (*arterial spin label*).

**Figure 1.**
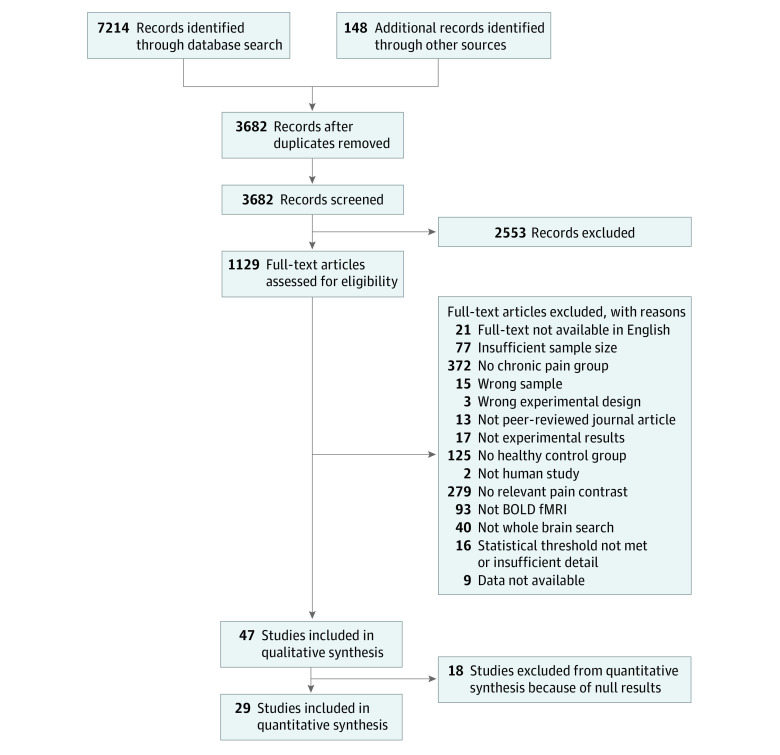
Preferred Reporting Items for Systematic Reviews and Meta-analyses (PRISMA) Flowchart Detailing Screening Process In total, 37 experiments from 29 articles were included in the meta-analysis.

Two independent reviewers (A.X. and B.L.) then evaluated titles and abstracts returned by this search for full-text screening. For full-text screening, we applied the inclusion criteria outlined below. This screening process resulted in a total of 58 experiments from 47 articles meeting inclusion criteria. Because neuroimaging meta-analyses only integrate significant results reported in individual experiments, only experiments reporting significant results were evaluated. This is a known limitation of neuroimaging meta-analyses (see Discussion section below). Accordingly, 37 experiments from 29 articles were included in the meta-analysis (Figure 1). More detail on our search and screening methods are in the eMethods in the Supplement.

### Inclusion Criteria

Experiments were only included if they met the following criteria, which adhered to recent neuroimaging meta-analysis guidelines^13,14^:

1. The experiment was from a peer-reviewed journal article written in English.
2. The experiment included a patient group with chronic pain and a healthy control group. Patients were considered to have chronic pain if they were formally diagnosed with a chronic pain condition (by a clinician, an assessment, or field-standard criteria) or reported experiencing pain for at least 6 months.
3. The experiment included at least 10 participants in the patient group and at least 10 participants in the healthy control group.
4. Participants were 18 years or older within both patient and healthy control groups.
5. All participants confirmed the noxious stimuli to be painful by explicitly reporting the stimuli being painful, by participant ratings of experienced pain from the stimuli, or by titrating the stimuli to a threshold predetermined to be painful by participants.
6. Brain responses to noxious stimuli were measured by task-activated, blood-oxygen level–dependent responses monitored with fMRI.
7. The experiment contained a within-participant “pain greater than baseline” contrast not confounded by other experimental manipulations (eg, pharmacological treatment before the pain induction).
8. The experiment contained a between-group “patients greater than healthy controls” contrast.
9. The field of view and reported results included the whole brain (ie, ROI analyses were excluded). This criterion was imposed to prevent bias toward a priori ROIs.
10. Results adequately corrected for multiple comparisons^9^ by either reporting activation at a voxel-level threshold of *P* < .001 (uncorrected) or a corrected cluster probability of *P* < .05. Articles with insufficient detail about their multiple comparisons correction methods were excluded.
11. Experimental results were reported in a standard, stereotaxic reference space coordinate system (Montreal Neurological Institute or Talairach space). If coordinate results were not reported in experiments that otherwise met inclusion criteria, we e-mailed corresponding authors and included the experiment if data were provided.

### Coordinate-Based Meta-analysis

Data were analyzed from December 2019 to February 2020. Activation likelihood estimation was used for all meta-analyses.^29,30,31^ More details on data extraction and the activation likelihood estimation algorithm are found in the eMethods in the Supplement. Adhering to current recommendations for type I error control, we applied a voxel-level threshold of *P* < .001 and cluster-level familywise error–corrected threshold of *P* < .05. To better understand the distribution of subthreshold results, we further examined unthresholded statistical maps.

Our primary analysis sought to identify areas associated with aberrant activity in response to noxious stimuli in patients with chronic pain by assessing differences between patients and controls, irrespective of the directionality of the association. If a publication had multiple related experiments from the same participants, we included all coordinates but treated them as a single experiment, thereby using only 1 set of coordinates per publication. Next, to assess whether group differences in response to noxious stimuli were influenced by differences in pain intensity, we separately analyzed experiments in which pain intensity was matched between groups based on subjective rating (18 experiments). We did not separately analyze experiments administrating a fixed intensity of stimulation across groups, because there was an insufficient number of experiments (11 experiments; current guidelines recommend at least 17).^27^ In addition, to evaluate the directionality of the response, we meta-analyzed experiments reporting greater brain activity in patients compared with controls (n = 23). We did not conduct separate analyses of experiments reporting less activity in patients than controls owing to an insufficient number of experiments (n = 14).^27^ Similarly, owing to an insufficient number of experiments, we did not subanalyze specific pain conditions.

Finally, we conducted a post hoc, nonpreregistered, ROI-based meta-analysis focused on brain regions known to be involved in pain processing, as identified by a pain network from a recent meta-analysis of pain responses in healthy volunteers.^32^ Briefly, this approach compares the sum of the activation likelihood estimation scores within the pain network with the sum of a null set of activation likelihood estimation scores generated from spatial permutations. Significance was determined by comparing the observed sum within the pain network with the null distribution using a threshold of *P* < .001. Further details are highlighted in the eMethods in the Supplement. All experiments, regardless of whether they reported greater or less activity in patients, were included in this ROI-based meta-analysis, whereas a subset of experiments reporting greater activity is reported in the eResults and eFigures 1 and 2 in the Supplement.

## Results

We meta-analyzed 37 experiments from 29 articles,^33,34,35,36,37,38,39,40,41,42,43,44,45,46,47,48,49,50,51,52,53,54,55,56,57,58,59,60,61^ including a total of 511 patients and 433 healthy controls (944 participants). Chronic pain conditions included migraines (n = 7), fibromyalgia (n = 5), irritable bowel syndrome (n = 5), chronic back pain (n = 4), complex regional pain syndrome (n = 2), chemotherapy-induced peripheral neuropathy (n = 1), osteoarthritis (n = 1), persistent dentoalveolar pain disorder (n = 1), posttraumatic headache (n = 1), vulvar vestibulitis (n = 1), and a mixed patient group (n = 1). Most of the experiments used mechanically induced pain (n = 14), whereas others used thermal (n = 10), electrical (n = 4), or chemically (n = 1) induced pain. Most experiments matched noxious stimuli in patients and controls based on similar perceptual ratings of pain intensity (n = 18). Remaining experiments (n = 11) induced pain at a fixed stimulus intensity across patients and control groups.

Eleven of the 29 experiments excluded the presence of any psychiatric comorbidities. Four experiments excluded participants based on the presence specific psychiatric comorbidities, such as anxiety, posttraumatic stress disorder, severe or unstable mental disorders, and current Axis I mental disorders. Two experiments explicitly did not exclude participants based on the criteria of psychiatric comorbidities, and 12 experiments did not provide criteria related to psychiatric comorbidities. More details on included experiments are present in the eTable in the Supplement. The original coordinate data and code for analysis can be found at https://github.com/PennLINC/Xu_fMRIChronicPain.

### Primary Meta-analysis

**Figure 2.**
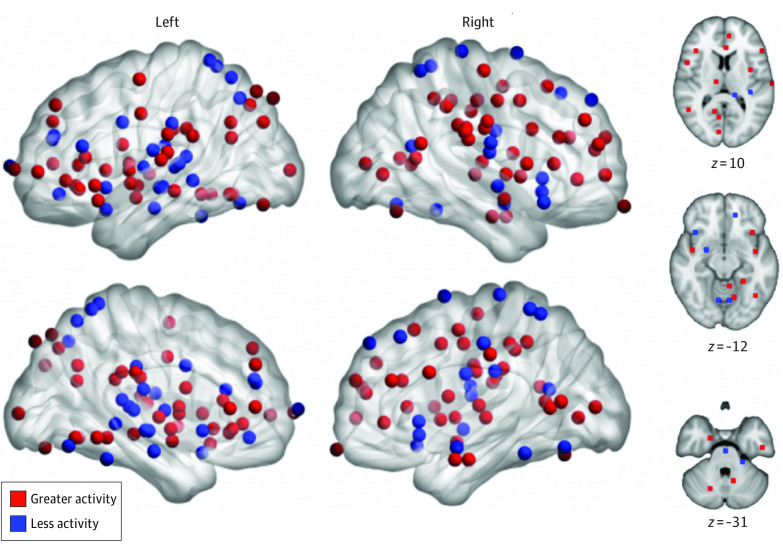
Distribution of Foci From Experiments Reporting Differences in Responses to Experimentally Induced Pain Between Patients With Chronic Pain and Healthy Controls Peak coordinates of clusters where activation was reported to be different between patients and controls had a broad spatial distribution. Red foci represent coordinates in experiments reporting greater pain responses in patients, whereas blue foci denote locations in experiments reporting reduced pain responses in patients.

Our primary meta-analysis evaluated whether convergent differences in responses to noxious stimuli between patients and healthy controls existed, regardless of the sign of the association. We did not find significant differences between groups at our preregistered statistical threshold (which included voxel height, *P* < .001 and familywise error–corrected cluster significance, *P* < .05). Unthresholded maps revealed a qualitatively wide distribution of foci reported across experiments (Figure 2); this lack of convergence was reflected by the null results.

**Figure 3.**
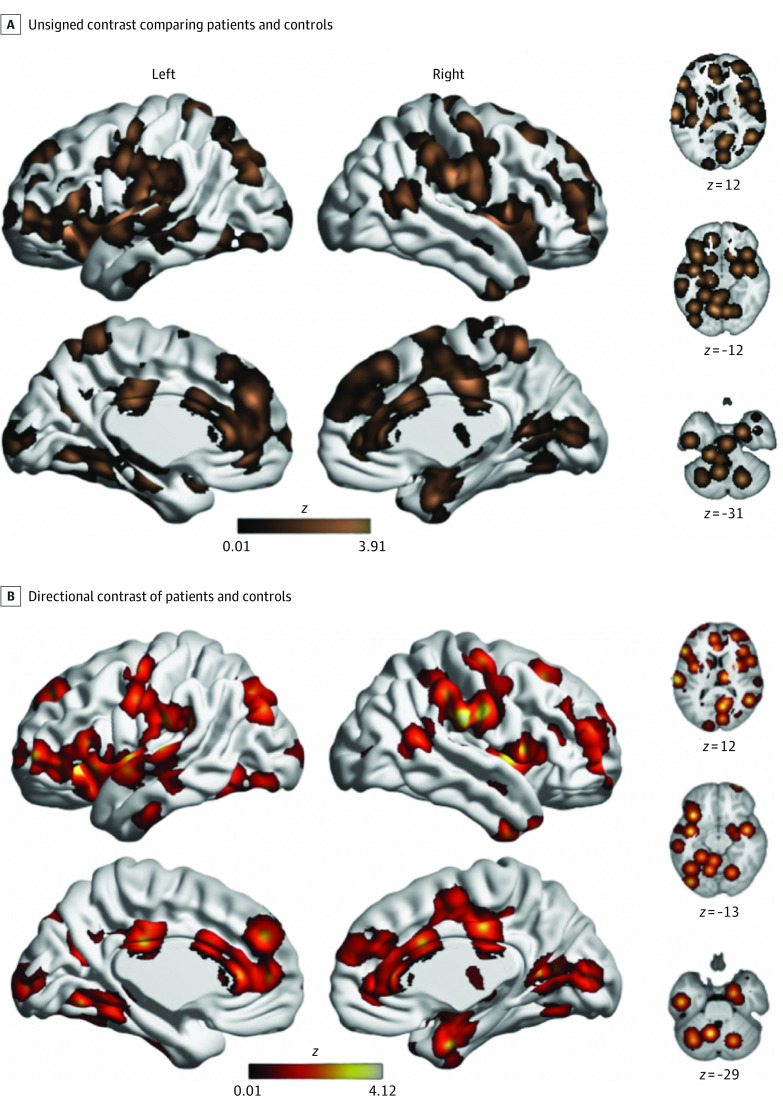
Unthresholded Maps of Differences Between Patients With Chronic Pain and Healthy Controls Maps display unthresholded associations from analyses. No between-group differences were significant at our preregistered statistical threshold (voxel height, *P* < .001; familywise error–corrected cluster significance, *P* < .05). However, pain-related regions appeared to be enriched for (nonsignificant) associations, which motivated post hoc regional analyses.

Subsequent analysis restricted to experiments matching subjective pain ratings across groups also did not reveal any significant differences (eResults and eFigures 1 and 2 in the Supplement). Similarly, separate analyses of experiments reporting greater brain activity with pain in patients did not reveal significant differences. However, unthresholded maps qualitatively suggested an enrichment of (nonsignificant) associations in pain-related brain regions (Figure 3). This observation motivated post hoc regional analyses.

### Post hoc Regional Analysis of Pain Network 

**Figure 4.**
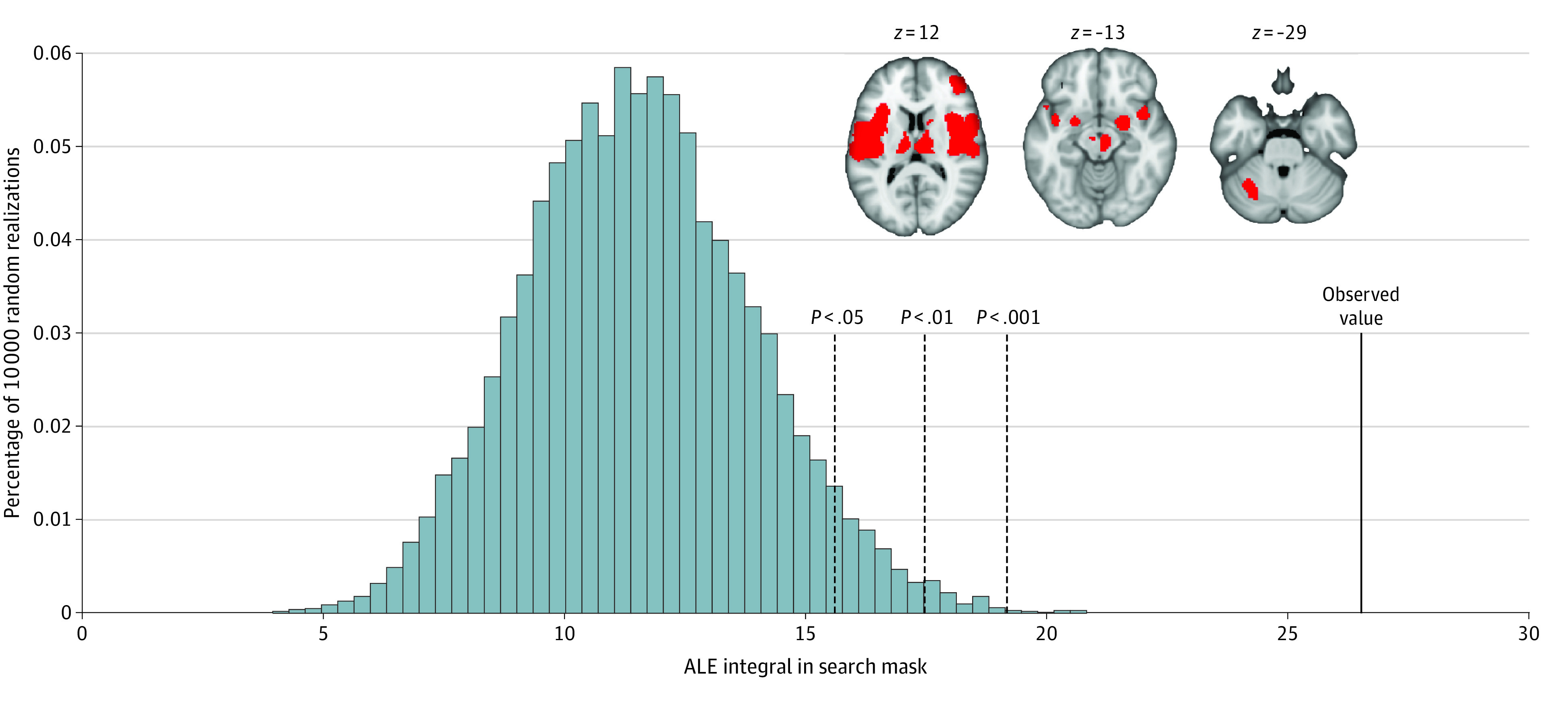
Post hoc Regional Analyses of Pain Network Histogram displays the distribution of the sum of activation likelihood estimation (ALE) scores within the pain network under the null distribution derived from spatial permutations. The observed value from our meta-analysis shows that our summed ALE score within the pain network exceeds that expected from the null distribution. This suggests a significant convergence of aberrant activity within the pain network in patients with chronic pain. Inset displays the pain network mask, defined by a prior meta-analysis of brain responses to pain in healthy volunteers.^32^

Based on examination of unthresholded maps, we conducted a post hoc, nonpreregistered regional analysis focused on the pain network, as defined by a previous meta-analysis examining brain responses to noxious stimuli in healthy volunteers.^32^ This regional analysis of unsigned associations revealed significant (*P* < .001) associations compared with the null distribution, suggesting that patients with chronic pain may have aberrant responses to noxious stimuli within the pain network (Figure 4). Follow-up analyses of directional associations revealed greater responses in patients than control individuals within this network (eResults and eFigures 1 and 2 in the Supplement).

## Discussion

Our findings suggest a lack of evidence for consistent differences in brain responses to noxious stimuli in patients with chronic pain. These null results were found in an adequate sample of experiments: 29 experiments were included in the meta-analysis, which is well-powered for a neuroimaging meta-analysis.^13,27^ However, post hoc, nonpreregistered regional analyses of responses within a previous meta-analytic map for pain^32^ revealed subtle differences in the aggregate activation of the pain network. This suggests that to the extent that differences in brain responses to noxious stimuli in chronic pain are present, they may be localized to core pain-processing areas. Notably, we did not set out to validate a specific model or theoretical framework in this study. Instead, we sought to resolve inconsistencies in the literature through application of rigorous methods. As such, this data-driven study is complementary to prior work that focuses on specific models of chronic pain.

These results align with 1 prior meta-analysis that similarly reported null results^14^ but stand in contrast to prior fMRI meta-analyses that have reported aberrant activity in a variety of regions, including cingulate cortex,^20,21,22^ insula,^20,21,22^ secondary somatosensory cortex,^20,21,22^ cerebellum,^21,22^ primary somatosensory cortex,^22^ prefrontal cortex,^22^ intraparietal lobule, thalamus,^22^ and supplementary motor area.^22^ Methodological differences, including differences in inclusion criteria and meta-analytic techniques, may have driven these disparate findings. In the present study, several important methodological choices that address 3 key issues bolster confidence in our results. First, we address the issue of conflating experimental design differences with differences between groups by focusing on within-experiment contrasts of brain responses to pain in patients compared with controls. Second, we address potential for regional bias by only including experiments reporting whole brain analyses. As a result of this inclusion criteria, we excluded 40 experiments because of use of a smaller search volume or using ROI-based approaches. Common regions examined include the primary somatosensory cortex, secondary somatosensory cortex, brainstem, anterior cingulate cortex, prefrontal cortex, thalamus, and insula.

Finally, we limited the potential for false-positive findings by requiring experiments to have at least 10 participants per group and to correct for multiple comparisons (with an uncorrected voxel height of *P* < .001 or cluster-corrected threshold of *P* < .05). These criteria are more rigorous than previous meta-analyses, which typically have not considered the type I error control of included experiments. Notably, this was by no means overly conservative: current standards recommend applying a voxel-height of *P* < .001 and a cluster-corrected threshold of *P* < .05. We did not require such a level of correction, because preliminary searches revealed that only a few experiments met that threshold or provided sufficient information regarding their methods. However, at the meta-analytic level, we applied these stringent standards for proper type I error control.^13,14,27^ As such, our approach aligns with the current best practices for avoiding spurious results.

Several factors may contribute to the observed null results. First, most of the included experiments matched noxious stimulation in patients and controls using subjective ratings of pain. This experimental design would pose difficulty in detecting other sources of abnormal brain responses to noxious stimuli in patients. For example, patients with central sensitization could show increased responses to noxious stimuli at lower levels of intensity compared with a control group, owing to amplification of pain from hyperalgesia or allodynia.^62^ Null differences can therefore be attributable to similar experiences of experimental pain induction that are unable to detect these differences between patients and controls.

Second, heterogeneity driven by differences in underlying neurobiology in chronic pain conditions,^16^ sample demographics,^63,64,65^ experimental design, and analytical pipelines^8,10,15^ may contribute to inconsistent results across experiments. Owing to the limited number of experiments meeting our inclusion criteria, we could not assess whether patient conditions with different etiology (ie, whether pain was a primary or secondary condition)^66^ and neurobiology^16^ differentially affect brain responses to noxious stimuli. Previous meta-analyses with less stringent inclusion criteria have shown different patterns of functional reorganization of the brain in different chronic pain conditions.^23^ These findings highlight the need to examine the influence of specific pain conditions on aberrant responses to pain perception when more experiments meeting rigorous inclusion criteria are available. For example, differences between visceral and nonvisceral pain, both at the level of pain perception and at the level of autonomic and emotional responses involvement, raise the possibility that patients with visceral pain show different brain responses to pain.^67^ Prior work^25^ has shown differences in brain areas involved in emotional arousal in patients with irritable bowel syndrome compared with healthy controls. Future studies should examine the possibility of these differences when more experiments are available.

Patients’ sex,^64^ age,^63^ and medication status^68^ may also play a role in their brain responses to pain. For example, patients’ sex may affect functional activity of sensorimotor regions, the insula, and emotional-arousal reactivity,^64^ whereas age may affect striatal pain modulatory mechanisms.^63^ However, an insufficient number of experiments included limited our ability to rigorously test the influence of these factors. Medication status, such as the use of opioids, nonsteroidal anti-inflammatory drugs, atypical antipsychotics, or antidepressants, may alter brain function in patients, but the diverse medication inclusion criteria used in each experiment creates difficulty in conducting subanalyses focused on medication status.^68^ Beyond patient condition and demographics, differences in pain stimulation also add to inconsistencies between reported brain regions across experiments. Prior work reporting differences in brain region responses to pain^20,23,69^ depend on stimulation location and modality (eg, thermal, mechanical), highlighting the need for further investigation.

Third, a known limitation of our meta-analytic algorithm is that experiments without significant results cannot be included. This limitation biases the meta-analysis toward finding significant results but also bolsters confidence in our null results, because we did not find significant associations even when null experiments were not included.

In contrast to these results, we qualitatively observed a nonsignificant enrichment of foci in pain-related regions in our unthresholded meta-analytic maps. Accordingly, we conducted exploratory regional analyses focused on a previously identified pain network^32^ and found that if differences in response to noxious stimuli between groups did exist, they are likely localized within the pain network. This exploratory analysis likely yielded significant results (whereas the main voxelwise analyses did not) because regional analyses do not require whole-brain multiple comparisons correction. Furthermore, regional analyses evaluate aggregate responses across the pain network rather than requiring precise spatial convergence, providing greater sensitivity that would only be necessary if aberrant responses were both subtle and regionally localized. Although exploratory and inconclusive, these results may motivate and constrain future studies.

### Limitations

This meta-analysis focused on differences in task-activated, blood-oxygen level–dependent responses to noxious stimuli between patients and healthy controls. However, our literature search also yielded related articles using other neuroimaging measures and experimental designs that may have differential sensitivity to chronic pain. Specifically, we did not evaluate measures such as functional connectivity,^70,71^ cerebral blood flow,^72^ differences between patients in the chronic vs subacute phase of pain,^73^ or patient responses to spontaneous pain.^74^

## Conclusions

This systematic review and meta-analysis quantitatively synthesizes studies reporting within-experiment comparisons between patients with chronic pain and healthy controls while adhering to best practices for neuroimaging meta-analyses. The preregistered approach did not find evidence of significant differences in brain responses to noxious stimuli in patients. However, exploratory, regional analyses suggested limited brain activation differences in canonical brain regions robustly activated in pain processing. Overall, the results suggest that, if abnormalities in brain responses to pain in chronic pain are present, their response patterns are likely not consistent or robust.

## Notes

Supplement. **eMethods.** Search, Screening, and Data Extraction **eResults.** Supplementary Results of Follow-up Subanalyses **eFigure 1.** Unthresholded Maps of Subanalyses of Experiments Matching Noxious Stimuli by Perceptual Rating **eFigure 2.** Post hoc Regional Analysis of Pain Network Suggest Greater Activity in Response to Pain in Patients With Chronic Pain **eTable.** Study Information About Articles and Experiments Included in Meta-analyses **eReferences.**
