# Supplementary material for: Brain Responses to Noxious Stimuli in Patients With Chronic Pain: A Systematic Review and Meta-analysis
Source: JAMA Netw Open. 2021 Jan 5;4(1):e2032236. doi: 10.1001/jamanetworkopen.2020.32236 (PMC7786252; doi:10.1001/jamanetworkopen.2020.32236)
Supplement: Supplement. — eMethods. Search, Screening, and Data Extraction eResults. Supplementary Results of Follow-up Subanalyses eFigure 1. Unthresholded Maps of Subanalyses of Experiments Matching Noxious Stimuli by Perceptual Rating eFigure 2. Post hoc Regional Analysis of Pain Network Suggest Greater Activity in Response to Pain in Patients With Chronic Pain eTable. Study Information About Articles and Experiments Included in Meta-analyses eReferences. [file jamanetwopen-e2032236-s001.pdf]

Supplementary Online Content

Xu A, Larsen B, Henn A, et al. Brain responses to noxious stimuli in patients with chronic pain: a systematic review and meta-analysis. *JAMA Netw Open*. 2021;4(1):e2032236. doi:10.1001/jamanetworkopen.2020.32236

eMethods. Search, Screening, and Data Extraction

eResults. Supplementary Results of Follow-up Subanalyses

eFigure 1. Unthresholded Maps of Subanalyses of Experiments Matching Noxious Stimuli by Perceptual Rating

eFigure 2. Post hoc Regional Analysis of Pain Network Suggest Greater Activity in Response to Pain in Patients With Chronic Pain

eTable. Study Information About Articles and Experiments Included in Meta-analyses

eReferences.

This supplementary material has been provided by the authors to give readers additional information about their work.

©2021 Xu A et al. *JAMA Network Open*.

eMethods. Search, Screening, and Data Extraction

Literature Search

We performed a literature search for neuroimaging experiments of experimentally induced pain in patients with chronic pain using both database searches and references cited in meta-analyses. The final literature search took place on May 28, 2019 and was restricted to articles published from 1990 to May 28, 2019.

The following standard literature databases were searched: PUBMED/MEDLINE, EMBASE, Web of Science, Cochrane Library, PsycINFO, and SCOPUS. We used the following search terms: ("fMRI" or "functional magnetic resonance imaging" or "BOLD" or "brain mapping") AND ("pain" or "noxious" or "nociception") AND ("patients" or "neuropathic" or "chronic pain" or "hyperalgesia" or "allodynia") OR ("arterial spin label"). When possible, we further filtered our search criteria to try to meet our inclusion criteria as much as possible by including only publications that were in English, that appeared in peer-reviewed journals (e.g., not conference papers), and that included experiments conducted in humans.

To identify potential candidate studies from reference lists, we also screened the resulting abstracts for meta-analyses related to pain. We then screened included studies within the meta-analysis for full text review. This resulted in reviewing the references from 14 meta-analyses.[30–43]

Screening

Two independent reviewers (AX, BL) were involved in the reviewing of the inclusion or exclusion of abstracts or full text screening. Abstracts were first assessed as to whether they included a physical pain contrast in healthy volunteers and whether they measured task-based blood-oxygen-level-dependent (BOLD) responses. In this stage of screening, we only excluded abstracts that explicitly mentioned (1) having a sample size of less than 10 subjects; (2) using only a neuroimaging modality that was not fMRI, such as EEG; or (3) only including animal experiments. Full text articles from included abstracts were then assessed for whether they met our inclusion criteria. Finally, at least two independent reviewers confirmed the decision for inclusion of articles marked for inclusion in the final analysis (AX, BL). In cases of reviewer decision disagreement, a senior third reviewer (TS) evaluated the article.

Data Extraction

Coordinates and information about each experiment were extracted manually by at least one author (AX or VS) and checked independently by another member of the study team (AX or VS). The following information about each paper was extracted for analyses: sample size; and whether the coordinate space was MNI or Talairach. If multiple related contrasts were present in an article, we included all contrasts but treated them as one experiment, thereby using only one set of coordinates in the meta-analysis per article. To provide more detail on experiments included in the meta-analysis, we also extracted more information about patients. This information included diagnosis, diagnosis method, duration of pain, medication status, modality of noxious stimulus (e.g., thermal, mechanical, chemical, or electrical), and location of induction.

Activation likelihood estimation

©2021 Xu A et al. *JAMA Network Open*.

We conducted meta-analyses using the coordinate-based meta-analytic method activation likelihood estimation (ALE) using a revised algorithm that allows for random effects inference.[44–47] Briefly, for each experiment included, ALE treats coordinates for the foci of reported clusters as the center of an uncertainty function modeled by a 3D Gaussian probability distribution. The full width at half-maximum (FWHM) of this 3D Gaussian kernel was determined by empirical data on between-subject and between-template (i.e., MNI or Talairach space coordinates) variance. Specifically, the algorithm takes into account between-subject variance by using a tighter Gaussian distribution for experiments with greater sample sizes to represent that these experiments should provide more reliable results of a true activation effect.[45] It also models uncertainty due to template use and transforms coordinates reported in Talairach coordinates into MNI coordinates.[48] This model then provides probabilities for all activation foci in each experiment, which were combined for each voxel, resulting in an individual modeled activation (MA) map for each experiment. By taking the union across all the MA-maps, we generated voxelwise ALE scores that describe the convergence of results at each particular location.[45] Note that MA-values reflect data for a single experiment while ALE-values integrate data across multiple experiments.

For ALE maps, the *p*-value was defined as the proportion of values obtained under a null distribution reflecting a random spatial association between experiments. The resulting non-parametric *p* values were subsequently thresholded using a voxelwise threshold of $p < 0.001$, reflecting current recommendations for best practices.[49,50] At this voxelwise threshold, the significance of cluster extent was estimated using 10,000 Monte-Carlo simulations; this distribution was calculated specifically for each meta-analysis conducted.[46,47] Clusters were considered significant if they achieved a family-wise corrected significance of $p < 0.05$. Prior to display, *p*-values were transformed into *z*-scores.

ROI-based analyses used an ROI mask constrained to regions activated by pain stimulation in healthy participants that was derived from a previous meta-analysis.[51] ROI-based meta-analyses resulted in an outcome measure of the sum of ALE scores across our mask (i.e., the ALE integral). This ALE integral was then compared with the sum of a null set of ALE scores generated from a random spatial distribution, which was estimated from 10,000 random realizations of the ALE integral in the search mask (thereby defining the null hypothesis). The ALE integral exceeding the sum of the null set would reflect whether the sum of ALE scores within the directed search mask were activated above chance.

©2021 Xu A et al. *JAMA Network Open*.

eResults. Supplementary Results of Follow-up Subanalyses

Sub-analyses of experiments matching noxious stimuli by perceptual rating

Sub-analyses of experiments matching noxious simulation to patients and controls by similar perceptual ratings of pain (n = 18) did not reveal any significant results (**eFigure 1**). There were not enough experiments matching noxious stimulation to patients and controls by similar stimuli intensity to conduct robust sub analyses (n = 11).

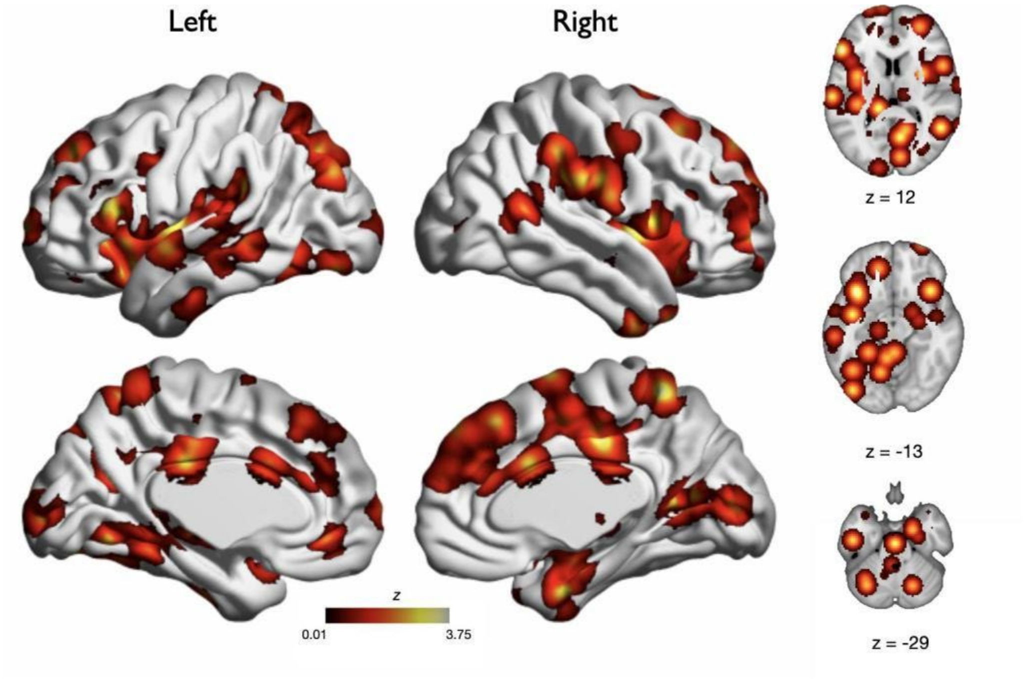

eFigure 1. Unthresholded Maps of Subanalyses of Experiments Matching Noxious Stimuli by Perceptual Rating

Maps display unthresholded effects of experiments matching noxious stimuli between groups by perceptual rating of pain. No between-group differences were significant at our pre-registered statistical threshold (voxel height $P < 0.001$, FWE-corrected cluster significance $P < 0.05$).

©2021 Xu A et al. *JAMA Network Open*.

Post-hoc regional sub-analysis of pain network show greater responses to pain in patients with chronic pain

Based on examination of unthresholded maps related to sub-analyses of experiments reporting greater activity in patients in response to pain ($n = 23$), we conducted post-hoc regional analyses focused on a previously reported pain network.[51] This ROI-based analysis revealed overall convergence of greater brain activity in patients in response to chronic pain. Sub-analysis of experiments reporting less activity in patients were not conducted due to an insufficient number of experiments to reliably meta-analyze.

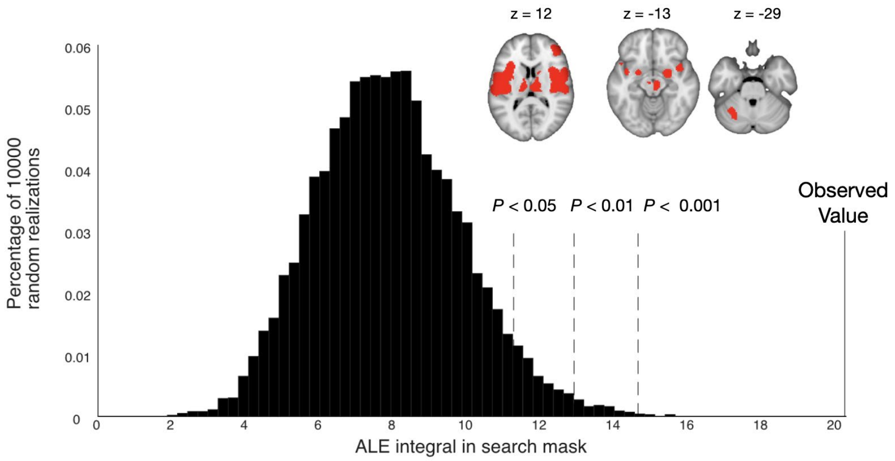

eFigure 2. Post hoc Regional Analysis of Pain Network Suggest Greater Activity in Response to Pain in Patients With Chronic Pain

Histogram shows distribution of summed ALE scores within the pain network under a null hypothesis of random, permuted spatial distribution. Inset displays the pain network masked used based on a prior meta-analysis of brain responses to pain in healthy volunteers.[51] Observed value from non-permuted data suggest a significant, overall convergence of greater responses to pain within the pain network in patient

©2021 Xu A et al. *JAMA Network Open*.

eTable. Study Information About Articles and Experiments Included in Meta-analyses

Description of sample, contrasts used, medication inclusion criteria, pain duration, stimulus modality and location, baseline used to measure against pain, method of matching noxious stimuli to patients and controls, and coordinates used. Citations of included experiments are listed in **eReferences**. IBS = irritable bowel syndrome, SE = standard error, SD = standard deviation, IQR = interquartile range

Article	N (Patients/ Controls)	Contrasts	Pain Condition	Medication inclusion criteria	Pain Duration Statistic	Pain Duration (Months)	Stimulus Modality	Stimulus Location	Baseline	Match	Coordinates Used
Leung 2016[1]	15/15	Controls > patients	Posttraumatic headache	Patients were asked not to take any headache-related medications on the day of scanning.	Range	36-108	Thermal	Left calf	Rest	Perceptual	Table 4
Freund 2010[2]	10/15	Patients > controls	Complex regional pain syndrome I	The usual medication directly before the scan (conducted at 8 o'clock in the evening) was postponed until the termination of the examination.	Range	1-32	Electrical	Left index finger	Rest	Perceptual	Table S4
Freund 2015[3]	10/15	Controls > patients	Complex regional pain syndrome I	The experiments took place at 8-o'clock in the evening with the patients not having taken their evening medication yet.	Range	1-32	Electrical	Left hand	Rest	Perceptual	Table 3

Article	N (Patients/ Controls)	Contrasts	Pain Condition	Medication inclusion criteria	Pain Duration Statistic	Pain Duration (Months)	Stimulus Modality	Stimulus Location	Baseline	Match	Coordinates Used
Mathur 2016[4]	14/14	Patients > controls; controls > patients	Migraine	All patients had taken medication in the past 24 h (e.g., antidepressant, NSAID, triptan, opioid, anticonvulsant, anxiolytic/muscle relaxant, other prophylactic, other analgesic).	Mean (SE) and range	130.8(24); 12-324	Thermal	Left forearm	Innocuous	Perceptual	Table 2
Gracely 2002[5]	16/16	Patients > controls; controls > patients	Fibromyalgia	Patients were allowed to continue taking any long-term medications, although analgesics were discontinued 12 hours before the baseline psychophysical evaluation and the fMRI sessions. Patients receiving opioid medications were excluded.	Not reported	Not reported	Mechanical	Left thumbnail	Innocuous	Perceptual	Table 3

©2021 Xu A et al. *JAMA Network Open*.

Article	N (Patients/ Controls)	Contrasts	Pain Condition	Medication inclusion criteria	Pain Duration Statistic	Pain Duration (Months)	Stimulus Modality	Stimulus Location	Baseline	Match	Coordinates Used
Bouhassira 2013[6]	20/11	Patients > controls	IBS	Patients discontinued analgesics, antispasmodics, laxatives and antidiarrheal agents at least 7 days before the start of experiment.	Mean (SD)	Patient group 1: 222 (112.8); Patient group 2: 219.6 (78)	Mechanical	Rectal	Rest	Perceptual	Table 2 (IBS patients vs healthy volunteers)
Boland 2014[7]	12/12	Patients > controls; controls > patients	Chemotherapy-induced peripheral neuropathy	Patients discontinue tricyclics, noradrenaline reuptake inhibitors and/or calcium channel blocker analgesic medication at least 48 hours prior to scanning; long term, stable dose of opioids.	Median [IQR]	24[10.8-38.4]	Thermal	Right foot and thigh	Rest	Perceptual	Figure 1b and 2b (coordinates are indicated in figure caption)
Pukall 2005[8]	14/14	Patients > controls	Vulvar vestibulitis	Sample excluded "regular medication use that could potentially interfere with cerebral blood flow."	Min required	6	Mechanical	Vulvar vestibule	Rest	Intensity	Table 3

©2021 Xu A et al. *JAMA Network Open*.

Article	N (Patients/ Controls)	Contrasts	Pain Condition	Medication inclusion criteria	Pain Duration Statistic	Pain Duration (Months)	Stimulus Modality	Stimulus Location	Baseline	Match	Coordinates Used
Elsenbruch 2010a[9]	15/12	Patients > controls	IBS	Sample excluded current use of psychiatric meditations.	Min required	12	Mechanical	Rectal	Rest	Perceptual	Table 2
Elsenbruch 2010b[10]	15/12	Patients > controls; controls > patients	IBS	Sample excluded medications known to affect the parameters of interest (e.g., neuroleptics, antipsychotics, beta-adrenergic medication).	Range	24-120+	Mechanical	Rectal	Rest	Perceptual	Supplementary table 2 (Health > IBS)
Burgmer 2012[11]	17/17	Patients > controls; controls > patients	Fibromyalgia	Medication altering pain perception or brain activation (e.g., pain medication, anxiolytics, antidepressants) were discontinued 48 h prior to functional scanning.	Min required	24	Mechanical	Right forearm	Rest	Intensity	Table 2
Moana-Filho 2015[12]	13/13	Patients > controls	Persistent dentoalveolar pain disorder	Not reported	Mean (SD)	96 (78)	Mechanical	Dental	Rest	Intensity	Table 4 & 5

©2021 Xu A et al. *JAMA Network Open*.

Article	N (Patients/ Controls)	Contrasts	Pain Condition	Medication inclusion criteria	Pain Duration Statistic	Pain Duration (Months)	Stimulus Modality	Stimulus Location	Baseline	Match	Coordinates Used
Baliki 2010[13]	16/16	Controls > patients	Chronic back pain	Not reported	Mean (SD)	94.2(82.68); Range 12-360	Thermal	Lower back	Rest	Intensity	Table S2 caption (text saying contrast)
Guleria 2017[14]	20/10	Patients > controls; controls > patients	IBS	Not reported	Median [IQR]	IBS-diarrhea: 60 [25.5-90]; IBS-constipation: 60 [33-120]	Mechanical	Rectal	Rest	Perceptual	Table 3
Hampson 2013[15]	48/13	Patients > controls	Multiple patients	Not reported	Mean (SD)	Vulvodynia: 58.8 (43.2); Fibromyalgia: 52.8 (54)	Mechanical	Vulva	Rest	Perceptual	Table 1 (Analysis 1)
Hiramatsu 2014[16]	12/11	Patients > controls	Knee osteoarthritis	Not reported	Min required	3	Electrical	Right knee	Innocuous	Perceptual	Table 2
Kim 2013[17]	21/11	Patients > controls	Fibromyalgia	Patients underwent routine clinical treatment using PGB.	Not reported	Not reported	Mechanical	Left thumbnail	Rest	Perceptual	Table 3
Lloyd 2008[18]	15/17	Patients > controls	Chronic lower back pain	Patients were allowed to continue on stable medication (e.g., opioids up to 60mg per day, paracetamol (acetaminophen) up to 4000 mg/day, and low doses of antidepressants and antiepileptic).	Mean (SD)	Waddell signs-high: 112 (86.7); Waddell signs-low: 123 (115)	Mechanical	Lumbar	Rest	Perceptual	coordinates reported in section "Between-Group fMRI Data"

©2021 Xu A et al. *JAMA Network Open*.

Article	N (Patients/ Controls)	Contrasts	Pain Condition	Medication inclusion criteria	Pain Duration Statistic	Pain Duration (Months)	Stimulus Modality	Stimulus Location	Baseline	Match	Coordinates Used
Dowdle 2019[19]	14/17	Patients > controls	Chronic pancreatitis	Patients were allowed to use chronic opiates (> 6 months).	Not reported	Not reported	Thermal	Left forearm	Rest	Perceptual	supplemental table 2
Jensen 2009[20]	16/16	Controls > patients	Fibromyalgia	Patients were washed out of all medications that could influence pain perception.	Not reported	Not reported	Mechanical	Left thumbnail	Innocuous	Perceptual	Table 3
Chen 2015[21]	15/20	Controls > patients	Migraine	Patients were weaned off analgesic drugs 1 week or longer, not in preventive treatment, and had not used any other drugs for at least 1 month prior to study.	Mean (SD)	With cutaneous allodynia 120.36(94.56); Without cutaneous allodynia 113.04(75.72)	Electrical	Left forearm	Rest	Intensity	Table 3 (contrast with controls--2 patient groups)
Rodriguez-Raecke 2014[22]	19/19	Patients > controls	Chronic lower back pain	Eight patients reported using antidepressant drugs as concomitant medication for pain (but not depression). Four patients used NSAID drugs. Two patients used opioids.	Not reported	Not reported	Thermal	Left forearm	Rest	Intensity	Table 1: Controls < CLBP

©2021 Xu A et al. *JAMA Network Open*.

Article	N (Patients/ Controls)	Contrasts	Pain Condition	Medication inclusion criteria	Pain Duration Statistic	Pain Duration (Months)	Stimulus Modality	Stimulus Location	Baseline	Match	Coordinates Used
Russo 2012[23]	16/16	Patients > controls; controls > patients	Migraine	All patients were both migraine-free and not taking attack medications for at least 3 days before MRI scan. They were also not taking any commonly prescribed medications for migraine prevention (e.g., valproate, topiramate, propranolol, amitriptyline, etc.).	Mean (SE)	111.36(60.48)	Thermal	Trigeminal	Rest	Intensity	Table 4
Russo 2017a[24]	16/16	Patients > controls	Migraine	Patients had not taken any acute migraine drug for at least 3 days before the scanning.	Mean (SE)	99.6(20.4)	Thermal	Cheek	Rest	Intensity	Figure 2A (pre-eTNS) -- ACC coordinate from results section
Russo 2017b[25]	20/20	Patients > controls; controls > patients	Migraine	Patients did not take rescue medications for at least three days before scanning.	Mean (SE)	With cutaneous allodynia: 142.8 (22.8); Without cutaneous allodynia: 171.6 (21.6)	Thermal	Trigeminal	Rest	Intensity	Table 2

©2021 Xu A et al. *JAMA Network Open*.

Article	N (Patients/ Controls)	Contrasts	Pain Condition	Medication inclusion criteria	Pain Duration Statistic	Pain Duration (Months)	Stimulus Modality	Stimulus Location	Baseline	Match	Coordinates Used
Russo 2019[26]	17/15	Patients > controls	Migraine	Patients did not take rescue medications for at least three days before scanning.	Mean (SE)	Without aura: 100.56 (19.92); With aura: 124.92 (26.88)	Thermal	Cheek	Rest	Intensity	Table 2
Stankewitz 2011[27]	20/20	Controls > patients	Migraine	No patients were taking medication for headaches.	Mean (SD)	152.4(97.2)	Chemical	Right nostril	Rest	Intensity	"Analyses of interictal migraine patients and matched controls"
Schmid 2015[28]	17/15	Patients > controls	IBS	Current use of systemic corticosteroids (>10 mg/day) or steroid treatment in higher doses within the preceding 8 weeks of the study were exclusionary.	Mean (SE)	54(44.4)	Mechanical	Rectal	Rest	Perceptual	Results (section "Adaptation phase")
Schreiber 2017[29]	38/15	Patients > controls	Fibromyalgia	Patients' medication regimens included gabapentin, a variety of antidepressants, nonsteroidal anti-inflammatory drugs, and acetaminophen, and were not altered during the course of this study.	Not reported	Not reported	Mechanical	Left calf	Rest	Perceptual	Table 4

©2021 Xu A et al. *JAMA Network Open*.

eReferences.

1. Leung A, Shukla S, Yang E, et al. Diminished supraspinal pain modulation in patients with mild traumatic brain injury. *Mol Pain*.2016;12. doi:10.1177/1744806916662661
2. Freund W, Wunderlich AP, Stuber G, et al. Different activation of opercular and posterior cingulate cortex (pcc) in patients with complex regional pain syndrome (crps i) compared with healthy controls during perception of electrically induced pain: A functional MRI study. *Clin J Pain*. 2010;26(4):339-347. doi:10.1097/AJP.0b013e3181cb4055
3. Freund W, Wunderlich AP, Stuber G, et al. The role of periaqueductal gray and cingulate cortex during suppression of pain in complex regional pain syndrome. *Clin J Pain*.2011;27(9):796-804. doi:10.1097/AJP.0b013e31821d9063
4. Mathur VA, Moayedi M, Keaser ML, et al. High frequency migraine is associated with lower acute pain sensitivity and abnormal insula activity related to migraine pain intensity, attack frequency, and pain catastrophizing. *Front Hum Neurosci*.2016;10(SEP2016). doi:10.3389/fnhum.2016.00489
5. Gracely RH, Petzke F, Wolf JM, Clauw DJ. Functional magnetic resonance imaging evidence of augmented pain processing in fibromyalgia. *Arthritis Rheum*.2002;46(5):1333-1343. doi:10.1002/art.10225
6. Bouhassira D, Moisset X, Jouet P, Duboc H, Coffin B, Sabate J-M. Changes in the modulation of spinal pain processing are related to severity in irritable bowel syndrome. *Neurogastroenterol Motil Off J Eur Gastrointest Motil Soc*.2013;25(7):623-e468. doi:10.1111/nmo.12123
7. Boland EG, Selvarajah D, Hunter M, et al. Central pain processing in chronic chemotherapy-induced peripheral neuropathy: A functional magnetic resonance imaging study. *PLoS ONE*. 2014;9(5). doi:10.1371/journal.pone.0096474
8. Pukall CF, Strigo IA, Binik YM, Amsel R, Khalifé S, Bushnell MC. Neural correlates of painful genital touch in women with vulvar vestibulitis syndrome. *Pain*.2005;115(1-2):118-127. doi:10.1016/j.pain.2005.02.020
9. Elsenbruch S, Rosenberger C, Enck P, Forsting M, Schedlowski M, Gizewski ER. Affective disturbances modulate the neural processing of visceral pain stimuli in irritable bowel syndrome: An fMRI study. Gut.2010;59(4):489-495. doi:10.1136/gut.2008.175000
10. Elsenbruch S, Rosenberger C, Bingel U, Forsting M, Schedlowski M, Gizewski ER. Patients with irritable bowel syndrome have altered emotional modulation of neural responses to visceral stimuli. Gastroenterology.2010;139(4):1310-1319.e4. doi:10.1053/j.gastro.2010.06.054
11. Burgmer M, Pfleiderer B, Maihöfner C, et al. Cerebral mechanisms of experimental hyperalgesia in fibromyalgia. *Eur J Pain Lond Engl*.2012;16(5):636-647.
12. Moana-Filho EJ, Bereiter DA, Nixdorf DR. Amplified brain processing of dentoalveolar pressure stimulus in persistent dentoalveolar pain disorder patients. *J Oral Facial Pain Headache*. 2015;29(4):349-362. doi:10.11607/ofph.1463
13. Baliki MN, Geha PY, Fields HL, Apkarian AV. Predicting Value of Pain and Analgesia: Nucleus Accumbens Response to Noxious Stimuli Changes in the Presence of Chronic Pain. *Neuron*. 2010;66(1):149-160. doi:10.1016/j.neuron.2010.03.002
14. Guleria A, Karyampudi A, Singh R, et al. Mapping of brain activations to rectal balloon distension stimuli in male patients with irritable bowel syndrome using functional magnetic resonance imaging. *J Neurogastroenterol Motil*.2017;23(3):415-427. doi:10.5056/jnm16148
15. Hampson JP, Reed BD, Clauw DJ, et al. Augmented central pain processing in vulvodynia. *J Pain*. 2013;14(6):579-589. doi:10.1016/j.jpain.2013.01.767
16. Hiramatsu T, Nakanishi K, Yoshimura S, et al. The dorsolateral prefrontal network is involved in pain perception in knee osteoarthritis patients. *Neurosci Lett*.2014;581:109-114. doi:10.1016/j.neulet.2014.08.027
17. Kim S-H, Lee Y, Lee S, Mun C-W. Evaluation of the Effectiveness of Pregabalin in Alleviating Pain Associated with Fibromyalgia: Using Functional

Magnetic Resonance Imaging Study. *PLoS ONE*.2013;8(9). doi:10.1371/journal.pone.0074099
18. Lloyd D, Findlay G, Roberts N, Nurmikko T. Differences in low back pain behavior are reflected in the cerebral response to tactile stimulation of the lower back. *Spine*.2008;33(12):1372-1377. doi:10.1097/BRS.0b013e3181734a8a
19. Dowdle LT, Borckardt JJ, Back SE, et al. Sensitized brain response to acute pain in patients using prescription opiates for chronic pain: A pilot study. *Drug Alcohol Depend*.2019;200:6-13. doi:10.1016/j.drugalcdep.2019.02.024
20. Jensen KB, Kosek E, Petzke F, et al. Evidence of dysfunctional pain inhibition in Fibromyalgia reflected in rACC during provoked pain. *Pain*.2009;144(1-2):95-100. doi:10.1016/j.pain.2009.03.018
21. Chen N, Zhang J, Wang P, Guo J, Zhou M, He L. Functional alterations of pain processing pathway in migraine patients with cutaneous allodynia. *Pain Med Malden Mass*.2015;16(6):1211-1220. doi:10.1111/pme.12690
22. Rodriguez-Raecke R, Ihle K, Ritter C, Muhtz C, Otte C, May A. Neuronal differences between chronic low back pain and depression regarding long-term habituation to pain. *Eur J Pain U K*. 2014;18(5):701-711. doi:10.1002/j.1532-2149.2013.00407.x
23. Russo A, Tessitore A, Esposito F, et al. Pain processing in patients with migraine: An event-related fMRI study during trigeminal nociceptive stimulation. *J Neurol*.2012;259(9):1903-1912. doi:10.1007/s00415-012-6438-1
24. Russo A, Tessitore A, Esposito F, et al. Functional changes of the perigenual part of the anterior cingulate cortex after external trigeminal neurostimulation in migraine patients. *Front Neurol*. 2017;8(JUN). doi:10.3389/fneur.2017.00282
25. Russo A, Esposito F, Conte F, et al. Functional interictal changes of pain processing in migraine with ictal cutaneous allodynia. *Cephalalgia Int J Headache*.2017;37(4):305-314. doi:10.1177/0333102416644969
26. Russo A, Tessitore A, Silvestro M, et al. Advanced visual network and cerebellar hyperresponsiveness to trigeminal nociception in migraine with aura. *J Headache Pain*. 2019;20(1):46. doi:10.1186/s10194-019-1002-3
27. Stankewitz A, Aderjan D, Eippert F, May A. Trigeminal nociceptive transmission in migraineurs predicts migraine attacks. *J Neurosci Off J Soc Neurosci*.2011;31(6):1937-1943. doi:10.1523/JNEUROSCI.4496-10.2011
28. Schmid J, Langhorst J, Gaß F, et al. Placebo analgesia in patients with functional and organic abdominal pain: A fMRI study in IBS, UC and healthy volunteers. *Gut*.2015;64(3):418-427. doi:10.1136/gutjnl-2013-306648
29. Schreiber KL, Loggia ML, Kim J, Cahalan CM, Napadow V, Edwards RR. Painful After-Sensations in Fibromyalgia are Linked to Catastrophizing and Differences in Brain Response in the Medial Temporal Lobe. *J Pain*.2017;18(7):855-867. doi:10.1016/j.jpain.2017.02.437
30. Sheehan J, Gaman A, Vangel M, Kuo B. "Pooled analysis of brain activity in Irritable Bowel Syndrome and controls during rectal balloon distension." *Neurogastroenterol Motil Off J Eur Gastrointest Motil Soc*.2011;23(4):336-e158. doi:10.1111/j.1365-2982.2010.01635.

©2021 Xu A et al. *JAMA Network Open*.

31. Lin C-S. Brain signature of chronic orofacial pain: a systematic review and meta-analysis on neuroimaging research of trigeminal neuropathic pain and temporomandibular joint disorders. *PloS One*. 2014;9(4):e94300. doi:10.1371/journal.pone.0094300
32. Ayoub LJ, Seminowicz DA, Moayedi M. A meta-analytic study of experimental and chronic orofacial pain excluding headache disorders. *NeuroImage Clin*.2018;20:901-912. doi:10.1016/j.nicl.2018.09.018
33. Friebel U, Eickhoff SB, Lotze M. Coordinate-based meta-analysis of experimentally induced and chronic persistent neuropathic pain. *NeuroImage*.2011;58(4):1070-1080. doi:10.1016/j.neuroimage.2011.07.022
34. Lanz S, Seifert F, Maihöfner C. Brain activity associated with pain, hyperalgesia and allodynia: An ALE meta-analysis. *J Neural Transm*.2011;118(8):1139-1154. doi:10.1007/s00702-011-0606-9
35. Solstrand Dahlberg L, Becerra L, Borsook D, Linnman C. Brain changes after spinal cord injury, a quantitative meta-analysis and review. *Neurosci Biobehav Rev*.2018;90:272-293. doi:10.1016/j.neubiorev.2018.04.018
36. Park J-Y, Kim Y-K, Kim S-Y, et al. Acupuncture modulates brain neural activity in patients: a systematic review and meta-analysis. *Orient Pharm Exp Med*.2017;17(2):111-126. doi:10.1007/s13596-017-0266-x
37. Boeckle M, Schrimpf M, Liegl G, Pieh C. Neural correlates of somatoform disorders from a meta-analytic perspective on neuroimaging studies. *NeuroImage Clin*.2016;11:606-613. doi:10.1016/j.nicl.2016.04.001
38. Tanasescu R, Cottam WJ, Condon L, Tench CR, Auer DP. Functional reorganisation in chronic pain and neural correlates of pain sensitisation: A coordinate based meta-analysis of 266 cutaneous pain fMRI studies. *Neurosci Biobehav Rev*.2016;68:120-133. doi:10.1016/j.neubiorev.2016.04.001
39. Tillisch K, Mayer EA, Labus JS. Quantitative Meta-analysis Identifies Brain Regions Activated During Rectal Distension in Irritable Bowel Syndrome. *Gastroenterology*.2011;140(1):91-100. doi:10.1053/j.gastro.2010.07.053
40. Jensen KB, Regenbogen C, Ohse MC, Frasnelli J, Freiherr J, Lundström JN. Brain activations during pain: a neuroimaging meta-analysis of patients with pain and healthy controls. *PAIN*. 2016;157(6):1279-1286. doi:10.1097/j.pain.0000000000000517
41. Dehghan M, Schmidt-Wilcke T, Pfleiderer B, et al. Coordinate-based (ALE) meta-analysis of brain activation in patients with fibromyalgia. *Hum Brain Mapp*.2016;37(5):1749-1758. doi:10.1002/hbm.23132
42. Apkarian AV, Bushnell MC, Treede R-D, Zubieta J-K. Human brain mechanisms of pain perception and regulation in health and disease. *Eur J Pain*.2005;9(4):463-484. doi:10.1016/j.ejpain.2004.11.001
43. Peyron R, Laurent B, García-Larrea L. Functional imaging of brain responses to pain. A review and meta-analysis (2000). *Neurophysiol Clin Neurophysiol*.2000;30(5):263-288. doi:10.1016/S0987-7053(00)00227-6
44. Turkeltaub PE, Eden GF, Jones KM, Zeffiro TA. Meta-analysis of the functional neuroanatomy of single-word reading: method and validation. *NeuroImage*.2002;16(3 Pt 1):765-780. doi:10.1006/nimg.2002.1131
45. Eickhoff SB, Laird AR, Grefkes C, Wang LE, Zilles K, Fox PT. Coordinate-based activation likelihood estimation meta-analysis of neuroimaging data: A random-effects approach based on empirical estimates of spatial uncertainty. *Hum Brain Mapp*.2009;30(9):2907-2926. doi:10.1002/hbm.20718
46. Eickhoff SB, Bzdok D, Laird AR, Kurth F, Fox PT. Activation likelihood estimation meta-analysis revisited. *NeuroImage*.2012;59(3):2349-2361. doi:10.1016/j.neuroimage.2011.09.017
47. Rottschy C, Langner R, Dogan I, et al. Modelling neural correlates of working memory: A coordinate-based meta-analysis. *NeuroImage*.2012;60(1):830-846. doi:10.1016/j.neuroimage.2011.11.050

©2021 Xu A et al. *JAMA Network Open*.

48. Lancaster JL, Tordesillas-Gutiérrez D, Martinez M, et al. Bias between MNI and Talairach coordinates analyzed using the ICBM-152 brain template. *Hum Brain Mapp*. 2007;28(11):1194-1205. doi:10.1002/hbm.20345
49. Eklund A, Nichols T, Knutsson H. Can parametric statistical methods be trusted for fMRI based group studies? *Proc Natl Acad Sci*.2016;113(28):7900-7905. doi:10.1073/pnas.1602413113
50. Eickhoff SB, Nichols TE, Laird AR, et al. Behavior, sensitivity, and power of activation likelihood estimation characterized by massive empirical simulation. *NeuroImage*.2016;137:70-85. doi:10.1016/j.neuroimage.2016.04.072
51. Xu A, Larsen B, Baller EB, et al. Convergent neural representations of experimentally-induced acute pain in healthy volunteers: A large-scale fMRI meta-analysis. *Neurosci Biobehav Rev*. 2020;112:300-323. doi:10.1016/j.neubiorev.2020.01.004

©2021 Xu A et al. *JAMA Network Open*.
